# A Continuous Procedure Based on Column Chromatography to Purify Anthocyanins from *Schisandra chinensis* by a Macroporous Resin plus Gel Filtration Chromatography

**DOI:** 10.3390/molecules21020204

**Published:** 2016-02-06

**Authors:** Daran Yue, Lei Yang, Shouxin Liu, Jian Li, Wei Li, Chunhui Ma

**Affiliations:** 1 College of Material Science and Engineering, Northeast Forestry University, Harbin 150040, China; yuedaran@163.com (D.Y.); liushouxin@126.com (S.L.); nefulijian@163.com (J.L.); 2 Key Laboratory of Forest Plant Ecology, Ministry of Education, Northeast Forestry University, Harbin 150040, China; ylmanefu@163.com; 3 Key Laboratory of Wood Science and Technology of Zhejiang Province, Zhejiang Agriculture and Forestry University, Hangzhou, Lin’an 311300, China

**Keywords:** *Schisandra chinensis*, anthocyanins, macroporous resin, gel filtration chromatography, LC-ESI-MS, antioxidant activities

## Abstract

In our previous study, as natural food colorants and antioxidants, the color and content stabilities of *Schisandra chinensis* (*S. chinensis*) anthocyanins were investigated. In this work, the purification process parameters of *S. chinensis* anthocyanins using a macroporous resin and gel filtration chromatography were evaluated. The optimized parameters of static adsorption and desorption were as follows. The selected resin is HPD-300 (nonpolar copolymer styrene type resin), and the anthocyanins adsorption saturation capacity of HPD-300 resin was 0.475 mg/g dry resin. Adsorption time was 4 h, and 0.517 mg/mL of *S. chinensis* anthocyanins was adsorbed on the resin column with a flow rate of 39 mL/h (3 BV/h). After adsorption, the anthocyanins were completely desorpted with 2.5 BV of 90% (*v*/*v*) ethanol solution, and the desorption flow rate was 13 mL/h (1 BV/h). After purification by dynamic adsorption and desorption, the anthocyanins content in the effluent increased from 47.6 mg/g to 128.4 mg/g, the purity of anthocyanins increased six-fold from 5.08% to 30.43%, and the anthocyanins recovery was 96.5%. The major constituent of *S. chinensis* anthocyanins was isolated with Bio-Gel P2 gel filtration chromatography, and it was detected by liquid chromatography electrospray ionisation tandem mass spectrometry (LC-ESI-MS) as cyanidin-3-*O*-xylosylrutinoside. Moreover, the antioxidant activities of *S. chinensis* anthocyanins were investigated. After purification using the HPD-300 resin, the antioxidant activities of anthocyanins were increased 1.2-fold (FRAP) and 1.7-fold (ABTS).

## 1. Introduction

Anthocyanins, an important source of natural colorants, have recently been applied in cosmetics, food, and pharmaceuticals because of the present trend towards replacing synthetic colorants [1]. Chemically, anthocyanins are glycosides of polyhydroxy and polymethoxy derivatives of 2-phenylbenzopyrylium or flavylium salts [2]. Anthocyanins as a group of flavonoid phenolic compounds are soluble in polar solvents such as acidified methanol, ethanol and water. Anthocyanins have numerous health beneficial properties, such as the prevention of heart disease, inhibition of carcinogenesis [3,4], anti-inflammatory activity [5], and antioxidant [6] and free-radical scavenging activities [7]. Therefore, there is an increased interest in the use of anthocyanins in functional food, nutraceutical and pharmaceutical industries.

*Schisandra chinensis* (Turcz.) Baill. (*S. chinensis*) fruits, used as a traditional medicinal herb and food additive, are widely distributed and cultivated in China, the Russian Far East, Korea and Japan [8]. Extensive studies have indicated that the major bioactive components of *S. chinensis* fruit are essential oils [9,10], biphenyl cyclooctene lignans [11,12,13], and anthocyanins [14].

Despite anthocyanins being found in many plant species, including strawberries [15], blueberries [16], blackberries [17] blackcurrants [15], black carrot [18], mulberries [19], red grapes [20], purple potatoes [21], red raspberries [22] and so on, there are almost no studies on purified *S. chinensis* anthocyanins using a macroporous resin. Moreover, compared to the conventional method, the macroporous resin enrichment is more effective because of its high efficiency, reduced solvent consumption, low cost, harmlessness to the environment, *etc*.

Recently, single separation technology has been unable to meet the high efficient production level. Therefore, the combination of chromatography technology received extensive attention of the researchers. The combined method of macroporous resin plus gel filtration chromatography in our study is investigated for purification of *S. chinensis* anthocyanins.

Therefore, in this work, the process parameters of purifying anthocyanins using a macroporous resin were investigated, and the main constituent of *S. chinensis* anthocyanins, which was isolated using Bio-Gel P2 gel filtration chromatography, was analyzed using liquid chromatography electrospray ionisation tandem mass spectrometry (LC-ESI-MS). Moreover, the antioxidant activities, including the total phenolic content, ferric reducing antioxidant power and free radical scavenging activity of *S. chinensis* anthocyanins (before and after purification with macroporous resin) were studied.

## 2. Results and Discussion

### 2.1. Static Adsorption and Desorption Tests of Anthocyanins

#### 2.1.1. Screening of Macroporous Resins

The appropriate macroporous resin was selected based on the capacity of adsorption and desorption, the ratio of desorption, and the adsorption speed. The adsorption capacity and the desorption capacity of all the resins tested in this study are shown in Table 1. Anthocyanins are polar compounds, which meant the polar resin had the highest adsorption ratio and a higher desorption ratio for anthocyanins. Among the tested resins, the nonpolar copolymer styrene type resin HPD-300 and HPD-5000 resins had the higher adsorption ratios (94.0% ± 0.6% for HPD-300 and 84.9% ± 0.6% for HPD-5000) and the HPD-200L and HPD-5000 had the higher desorption ratios (87.2% ± 0.7% for HPD-200L and 84.6% ± 0.6% for HPD-5000) for anthocyanins. This indicated that a bigger average pore diameter of resin aided desorption. However, the selectivity of a single solute in multi-solute system decreased because of the increase in average pore diameter, which resulted in a high desorption ratio but low adsorption and desorption capacities [23]. In order to comprehensively evaluate the adsorption ratio and desorption ratio, HPD-300, HPD-5000 and HPD-200L were used for the adsorption and desorption kinetics tests.

#### 2.1.2. Adsorption and Desorption Kinetics Curves

**Figure 1:**
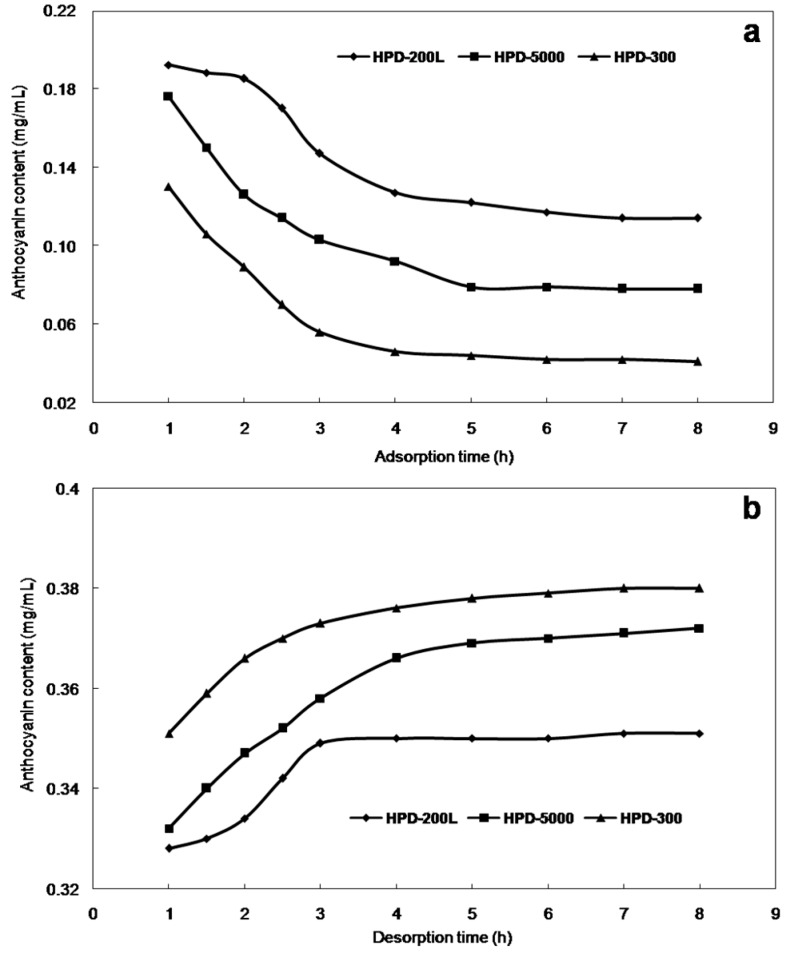
The adsorption (**a**) and desorption (**b**) kinetic curves for *S. chinensis* anthocyanins on HPD-300 resin.

The adsorption and desorption kinetics curves for anthocyanins on HPD-300, HPD-5000 and HPD-200L are shown in Figure 1. The adsorption capacity of anthocyanins increased with adsorption time and tended to equilibrate at about 5 h (Figure 1a). In the first 3 h, the adsorption capacity increased slowly, and between 3 and 5 h it increased rapidly, while after 5 h the slope of curve indicated equilibrium was reached. The desorption ratio of anthocyanins increased with desorption time, and reached equilibrium at about 4 h (Figure 1b). A comprehensive evaluation of the adsorption-desorption ratio and kinetics curves for anthocyanins indicated that HPD-300 was the best resin for purifying the *S. chinensis* anthocyanins.

**Table 1:** Physical properties and static adsorption-desorption characteristics of the test macroporous resins.

Trade Name	Polarity ^a^	Surface Area ^a^ (m^2^/g)	Average Pore Diameter ^a^ (nm)	Moisture Contents (%)	*D_e_* of Anthocyan in ^b^ (%)	*D_d_* of Anthocyan in ^b^ (%)
HPD-100	Non-polar	650–700	85–90	65.00 ± 1.85	81.4 ± 0.5	70.8 ± 0.5
HPD-100A	Non-polar	650–700	95–100	66.67 ± 1.01	36.8 ± 0.6	67.4 ± 0.6
HPD-300	Non-polar	800–870	80–85	75.52 ± 1.77	94.0 ± 0.6	79.9 ± 1.2
HPD-700	Non-polar	650–700	85–90	66.10 ± 1.31	59.4 ± 0.2	65.5 ± 0.6
HPD-5000	Non-polar	550–600	100–110	73.28 ± 1.32	84.9 ± 0.6	84.6 ± 0.6
AB-8	Weak-polar	480–520	130–140	65.00 ± 1.24	66.2 ± 0.3	81.9 ± 0.8
D101	Weak-polar	400–600	100–120	66.47 ± 1.62	77.4 ± 0.9	72.5 ± 0.8
HPD-400	Polar	500–550	75–80	68.93 ± 1.73	70.8 ± 0.8	80.6 ± 1.1
HPD-200L	Polar	500–550	80–90	72.86 ± 1.33	77.8 ± 1.1	87.2 ± 0.7
HPD-400A	Polar	500–550	85–90	66.48 ± 1.64	62.7 ± 1.2	72.8 ± 0.5
HPD-450	Polar	500–550	90–110	72.00 ± 1.58	53.6 ± 2.0	67.9 ± 1.0
HPD-750	Polar	650–700	85–90	57.58 ± 1.87	50.7 ± 1.1	63.4 ± 0.9
HPD-500	Strong-polar	500–550	55–75	70.45 ± 1.77	32.5 ± 0.4	62.5 ± 1.7
HPD-600	Strong-polar	550–600	80–90	69.32 ± 1.75	32.3 ± 0.4	62.9 ± 0.4
HPD-850	Strong-polar	1100–1300	85–95	46.81 ± 1.44	33.8 ± 0.8	87.4 ± 0.6

^a^ Parameters in the table provided by manufacturer of the resins. ^b^ In the static absorption and desorption experiment, 1.0 g adsorbent (dry resin weight) together with 50 mL of extract solution were added into a flask, shaken (100 rpm) for 8 h at 25 °C. After adsorption, the resins were washed with 50 mL deionized water and then static desorption was also performed in the shaker at 25 °C for 8 h. The process was repeated three times.

#### 2.1.3. Adsorption Temperature

**Figure 2:**
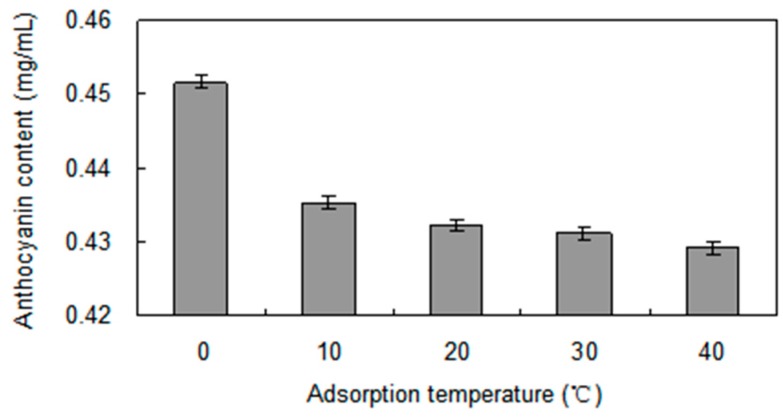
Effect of absorption temperature on *S. chinensis* anthocyanins content with HPD-300 resin.

Adsorption tests of anthocyanins on the HPD-300 resin were performed at four different temperatures (Figure 2). With the same initial concentration of anthocyanins, the adsorption capacities decreased as the temperature increased from 0 to 40 °C, and the adsorption was slower than desorption, which implied that the adsorption process was exothermic. This indicated that the adsorption process should be carried out at lower temperatures. Therefore, 0 °C was selected as the adsorption temperature.

#### 2.1.4. Adsorption Isotherms

The parameters of adsorption isotherms were obtained for anthocyanins (Table 2) with an initial anthocyanins concentration of 0.474 mg/mL.

**Table 2:** Parameters of adsorption isotherms.

T ^a^ (°C)	*C_i_* ^b^ (mg/mL)	*C_e_* ^b^ (mg/mL)	ns ^c^ (mg/mg)	ns*/C_e_* (L/g)	ln *C_e_*	ln ns	ln ns/ln *C_e_*
0	0.119	0.005	0.0057	1.14	−5.298	−5.167	0.98
0	0.237	0.014	0.01115	0.796	−4.269	−4.496	1.05
0	0.474	0.033	0.02205	0.668	−3.411	−3.814	1.12
0	0.948	0.077	0.04355	0.565	−2.564	−3.134	1.22
0	1.896	0.211	0.08425	0.399	−1.556	−2.474	1.59

^a^ T is the operation temperature of static adsorption test. ^b^ *C_i_* and *C_e_* are the initial and equilibrium concentrations of anthocyanin, respectively. ^c^ ns is the apparent adsorption quantity.

**Figure 3:**
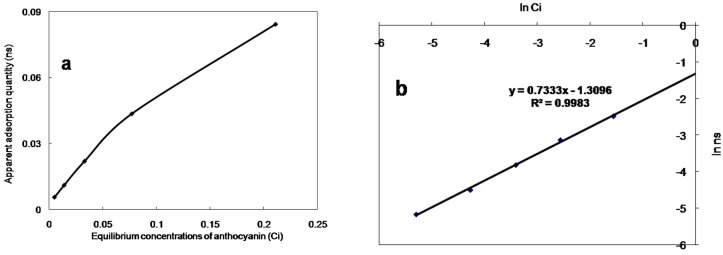
Langmuir adsorption isotherms (**a**) and Freundlich adsorption isotherms (**b**) of *S. chinensis* anthocyanins on HPD-300 resin.

The equations and Langmuir and Freundlich correlation coefficients are summarized in Figure 3. The Langmuir equation assumes uniform adsorption on the surface of the macroporous resin, and that there was no force between the adsorbent molecules. It only applies to mono molecule layer adsorption. However, the Freundlich equation was derived according to the uneven adsorption on the surface of the macroporous resin hypothesis, and has been combined with the Langmuir adsorption model. The correlation coefficients of the Langmuir and Freundlich equations at 0 °C were 0.9805 and 0.9983, respectively. The Freundlich equation provided the best description of the anthocyanins adsorption behaviour on the HPD-300 resin. The results indicated that the adsorption process was not a mono molecule layer adsorption. By a linear equation lg ns = lg a + 1/*n ×* lg *Ci*, and a = 0.27, 1/*n* = 0.7333. Therefore, the Freundlich equation was ns = 0.27*Ci*^0.7333^.

### 2.2. Dynamic Adsorption and Desorption Tests of Anthocyanins

#### 2.2.1. Dynamic Leakage Curves

**Figure 4:**
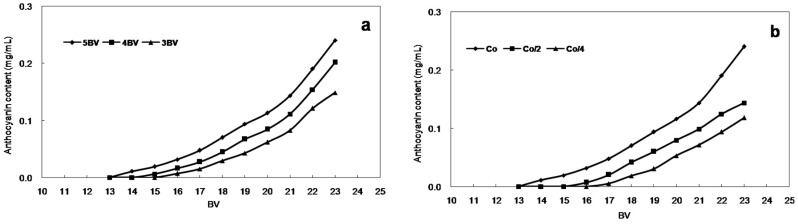
Leakage curves with (**a**) different current velocity and (**b**) different sample concentrations.

When the maximum quantity of solute has adsorbed on the resin, adsorption decreases or is balanced, and the solute desorbs from the resin. Consequently, it is important to construct leakage curves to evaluate the quantity of resin, the volume of sample solution, and the appropriate sample flow rate. The dynamic leakage curves obtained on HPD-300 resin were based on the volume of eluent and the flow rate. The results are shown in Figure 4a for the lower flow rate and the lower leakage (*C*_0_ = 0.47 mg/mL). At the lowest flow rate of 3 BV/h, the best adsorption performance was obtained, which is likely because of better particle diffusion in the sample solutions. However, the lower flow rate increased the experimental time. Despite this, 3 BV/h was chosen as the most appropriate sample flow rate for further experiments. Under this condition, the leakage volume of sample solution was approximately 18 times the BV (234 mL). In Figure 4b, the dynamic leakage curves shown for HPD-300 resin were based on the concentration of anthocyanins in the initial sample solution: the higher the concentration of anthocyanins, the faster the leakage. Therefore, the absorption action was easier at a lower concentration of absorbate solution. When the concentration of anthocyanins was *C*_0_/4, the leakage volume was approximately 20 times the BV (260 mL).

#### 2.2.2. Dynamic Desorption Curves

**Figure 5:**
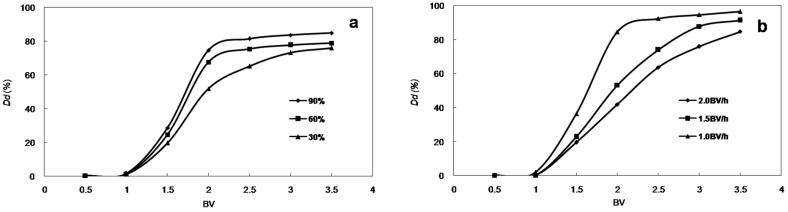
Dynamic desorption curves with (**a**) different ethanol volume fractions and (**b**) different current velocities (the ordinate is desorption ratio of anthocyanins).

Dynamic desorption curves on HPD-300 resin were obtained based on the volume and flow rate of the desorption solution. Ethanol-water solutions with different volume fractions (30:70, 60:40, and 90:10, *v*/*v*) were used for the desorption tests. As the ethanol volume fraction increased, the desorption ratio increased rapidly (Figure 5a). To ensure the efficiency and economy of the process, ethanol–water (90:10, *v*/*v*) solution was selected as the desorption solution and used in the dynamic desorption experiment.

The flow rates investigated in this test were 1, 1.5 and 2 BV/h. At the flow rate of 1 BV/h, anthocyanins were totally desorbed with a solvent volume of 2.5 BV (Figure 5b). By comparison, at the flow rates of 1.5 and 2 BV/h, anthocyanins were totally desorbed with a solvent volume of 3 BV. These results indicate that the lower desorption flow rate produced the most concentrated product among the flow rates tested. Therefore, this flow rate is better for the adsorption in terms of lower solvent use and high efficiency.

**Figure 6:**
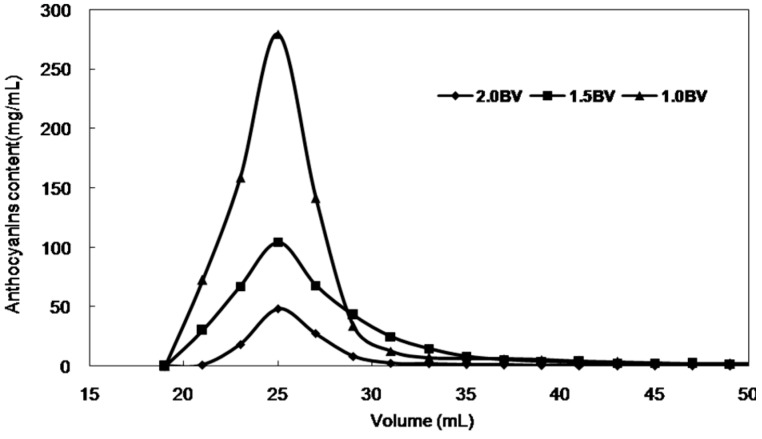
Dynamic desorption curves with different current velocities (the ordinate is the desorption content of anthocyanins).

The dynamic adsorption and desorption results and optimized parameters can be summarized as follows. For adsorption, the concentration of anthocyanins in the sample solution was 0.474 mg/mL. The processing volume was 260 mL (20 BV), and the flow rate was 3 BV/h. It was then washed with 3 BV deionized water at a flow rate of 3 BV/h. For desorption, the elution solvent was 32.5 mL (2.5 BV) ethanol–water (90:10, *v*/*v*) at a flow rate of 1 BV/h. After purification using the HPD-300 resin, samples were combined, concentrated in a rotary evaporator, and dried under vacuum. The purity of anthocyanins increased six-fold from 5.08% to 30.43%, and the anthocyanins recovery was 96.5%. The dynamic desorption curves with different flow rates are shown in Figure 6.

### 2.3. Gel Filtration Chromatography

**Figure 7:**
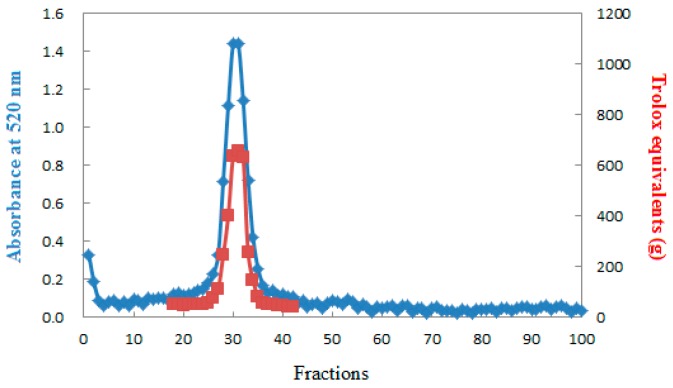
Gel filtration chromatography purification profile for *S. chinensis* anthocyanins after macroporous resin chromatography.

About 400 mL of anthocyanins solution, obtained by a scale-up experiment using macroporous resin was subjected to molecular exclusion chromatography, and a total of 100 fractions were collected (Figure 7). The gel-filtration resin used was Bio-Gel P2, and the recycle yield of anthocyanins (fractions 18–42) was 89.24%. The results showed that the plus gel filtration chromatography is efficient for purification of anthocyanins from *S. chinensis* fruits as well as from other plant.

Figure 7 shows fractions that had ferric reducing antioxidant power (fractions 18–42). After the 45th fraction, non-ferric reducing antioxidant power was observed corresponding to non-targets polyphenols. Comparison with previous research results, on the content and color stability of *S. chinensis* anthocyanins [14] and the oxidation stabilities under different external conditions, were also investigated (The research results will be published in other article).

### 2.4. LC-MS Analysis of S. chinensis Major Anthocyanins

**Figure 8:**
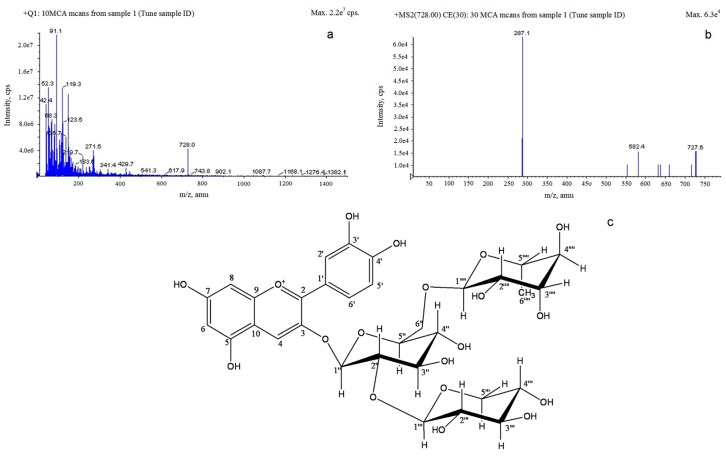
LC-MS chromatograms of Cyanidin-3-*O*-xylosylrutinoside: (**a**) MS1 chromatograms of cyanidin-3-*O*-xylosylrutinoside under ESI-MS in positive mode; (**b**) MS2 chromatograms of cyanidin-3-*O*-xylosylrutinoside under ESI-MS in positive mode; and (**c**) Proposed structure of cyanidin-3-*O*-xylosylrutinoside as an anthocyanin in *S. chinensis*.

The effluent liquid obtained from gel-filtration resin was concentrated under vacuum to dryness and then dissolved in 10% methanol, and used for the structure determination of *S. chinensis* fruit anthocyanins by LC-ESI-MS (Figure 8).

A major Liquid chromatographic peak of *S. chinensis* anthocyan represented about 95% of the total absorbable compounds at 520 nm, and the mass-to-charge ratio (*m*/*z*) and molecular weight of the major anthocyan was determined to be 727 [24]. As a result, it was confirmed that the relative intensity of the cyanidin-3-*O*-xylosylrutinoside (Cya-3-*O*-xylrut; *m*/*z* 727) decreased with an increase in fragmentor voltage, whereas the *m*/*z* 287.1 assigned to cyanidin was generated. Moreover, as can be seen in Figure 8b, another fragmented ion molecule (*m*/*z* 582.4) was detected and its molecular weight corresponded exactly with [M − rhamnose]^+^. The β-(1,6) linkage between glucose and rhamnose residues may be weaker than other glycosidic bonds in Cya-3-*O*-xylrut, which thus resulted inCya-3-*O*-rut during fragmentation. According to a few studies, Cya-3-*O*-xylrut was one of the major anthocyanins in red currant [25], and the structure of Cya-3-*O*-xylrut is shown in Figure 8e [26]. Thus, *S. chinensis* may be a unique source of highly pure Cya-3-*O*-xylrut.

### 2.5. Antioxidant Activities of Anthocyanins

#### 2.5.1. Determination of Total Phenolic Content

The radical scavenging ability and antioxidant activities of phenolic compounds are because of their hydroxyl groups [27]. As shown in Table 3, the total phenolic content of anthocyanins before purification with HPD-300 was 116.55 (mg/g catechins equivalents). The total phenolic content of anthocyanins after purification with HPD-300 was 519.55 (mg/g catechins equivalents). In short, the total phenolic content of *S. chinensis* anthocyanins consisting exclusively of Cya-3-Oxylrut increased 4.5-fold.

#### 2.5.2. Ferric Reducing Antioxidant Power (FRAP)

The reduction capacity of a compound may serve as a significant indicator of its potential antioxidant activity [28]. A higher absorbance indicated a higher ferric reducing power. As displayed in Table 3, the reducing power of the sample before purification with HPD-300 was 754.05 (g Trolox equivalents) and 902.05 (g Trolox equivalents) after purification with HPD-300. In brief, the reducing power of *S. chinensis* anthocyan consisting exclusively of Cya-3-Oxylrut increased 1.2-fold.

#### 2.5.3. Free Radical Scavenging Activity (ABTS)

From Table 3, the contribution of anthocyanins on the extract’s total ABTS radical scavenging activities was determined to be 368.727 (g Trolox equivalents) before purification with HPD-300, and 631.455 (g Trolox equivalents) after purification with HPD-300. Therefore, our results suggest that *S. chinensis* anthocyanins consisting exclusively of Cya-3-Oxylrut increased 1.7-fold.

**Table 3:** Results of antioxidant activity tests.

Tests	Before purification with HPD-300	After Purification with HPD-300
Concentration of anthocyanin (mg/mL)	0.474	1.247
Purity of anthocyanin (%)	5.08	30.43
Total phenolic content of anthocyanins (mg/g catechins equivalents)	116.55	519.55
Ferric reducing antioxidant power of anthocyanins (TE/g)	754.050	902.050
Free radical scavenging activity of anthocyanins (TE/g)	368.727	631.455

## 3. Experimental Section

### 3.1. Materials

*S. chinensis* fruit was purchased from San Keshu Trading (Heilongjiang, China) and identified by Prof. Shao-quan Nie from the Key Laboratory of Forest Plant Ecology, Northeast Forestry University. The same batch of sample was used in these experiments.

Folin–Ciocalteu’s reagent, 2,2′-azinobis-(3-ethylbenzothiazoline-6-sulfonic acid) (ABTS, 95%), and 6-hydroxy-2,5,7,8-tetramethylchromane-2-carboxylic acid (Trolox) were purchased from Sigma-Aldrich (St. Louis, MO, USA).

All the reagents obtained from Beijing Chemical Reagents Co. (Beijing, China) were of analytical grade. Deionized water was purified by a Milli-Q water purification system from Millipore (Bedford, MA, USA). Acetonitrile and acetic acid of HPLC grade were purchased from J & K Chemical Ltd. (Beijing, China), and macroporous resins were purchased from Guangfu Fine Chemical Research Institute (Tianjin, China).

### 3.2. Methods

#### 3.2.1. Preparation of *S. chinensis* Anthocyanins Extracts

*S. chinensis* fruits (150.0 g) were sieved using a 40–60 mesh after crushing, and then extracted with stirring using 1500 mL 1.0 M hydrochloric acid [14]. The supernatant fluid after filtration was extracted two times with the same volume of petroleum ether, then the fat-soluble impurities were removed, and extracted two times with the same volume of ethyl acetate, and then the water layer was used for future purification and antioxidant experiments.

#### 3.2.2. Static Adsorption and Desorption Tests

One gram of macroporous resin (dry weight) with 50 mL of the anthocyanins solution, which was extracted as described in Section 3.2.1, was shaken in a table concentrator at 100 rpm for 8 h at 25 °C. After adsorption, the resin was washed with 50 mL of deionized water, and then static desorption was performed with 50 mL of ethanol solution by shaking at 25 °C for 8 h. The process was repeated three times for each set of conditions. Adsorption temperature curves on the HPD-300 resin were tested at 0, 10, 20, 30 and 40 °C, respectively. Adsorption isotherms on the HPD-300 resin were tested with sample concentrations of 4*C*_0_, 2*C*_0_, *C*_0_, 0.5*C*_0_, and 0.25*C*_0_ (*C*_0_ = 0.474 mg/mL, which was the initial concentration of anthocyanins in the sample solution).

#### 3.2.3. Dynamic Adsorption and Desorption Tests

Dynamic adsorption and desorption experiments were carried out using a fixed-bed column separator (15 mm × 300 mm) wet-packed with 5.0 g of dry HPD-300 resin. The height of the resin bed was about 12.5 cm and the bed volumes (BV) of resin were approximately 13 mL. The crude extract solution was pumped through the fixed-bed at 39 mL/h, which was 3 BV/h for HPD-300. When the adsorption reached equilibrium, the fixed-bed column separator was washed with 39 mL of distilled water (3 BV), and then 32 mL (2.5 BV) of ethanol–water (90:10, *v*/*v*) at room temperature was injected into the fixed-bed column separator at 13 mL/h (1 BV/h) by a metering pump.

#### 3.2.4. Regeneration of Resins

First, the exhausted macroporous resins were soaked with 95% ethanol solution (1.0 g dry resin: 25 mL 95% ethanol solution), and then washed successively with 1 mol/L NaOH (10 BV) and 1 mol/L HCl (10 BV). Finally, the resins were washed with deionized water until the pH was about 7.

#### 3.2.5. Gel Filtration Chromatography

About 400 mL of anthocyanins solution, obtained by a scale-up experiment on the macroporous resin, were subjected to molecular exclusion chromatography. A glass column with a 15 mm ID and 300 mm length was packed with Bio-Gel P2andequilibrated with one and a half column volumes of acetic acid. The sample was then eluted with acetic acid, pH 2.5 at flow rate of 0.8 mL/min, and elution sample volumes (4 mL/tube) were collected using a fraction collector and were monitored by a spectrophotometer at 520 nm (Shimadzu 160 spectrophotometer). The column was then washed with acetic acid, pH 2.5 until no further anthocyanins were eluted. Anthocyanin-rich fractions were identified and concentrated under vacuum prior to further identification.

#### 3.2.6. LC-ESI-MS Analysis Method

An Agilent 1100 series HPLC system equipped with a G1312A Bin pump and G1379A Degasser (Agilent, San Jose, CA, USA), and a G1316A automatic column temperature control box and 2487 UV-detector (Waters, Boston, MA, USA) was used. Chromatographic separation was performed on a HiQ sil-C18 reversed-phase column (4.6 mm × 250 mm, 5 µm, KYA Technologies Corporation, Tokyo, Japan). Three percent acetic acid solution was used as eluent A, and HPLC-grade acetonitrile was used as eluent B. The gradient profile began at 10%–15% B at 40 min, 20% B at 45 min, and subsequently at an isocratic flow with 20% B for 20 min, and then returned to initial conditions at 65 min and kept for5 min. The flow rate was 1.0 mL/min, and the column temperature was maintained at 25 °C. The injection volume was 10 µL, and the detection wavelength was 520 nm.

An API3000 Triple tandem quadrupole mass spectrometer with a Turbolon-Spray interface from Applied Biosystems (Applied Biosystems, Foster City, CA, USA) was operated in positive electro spray ionization (ESI+, *m*/*z* 100–1000) source mode. All mass spectra were acquired in multiple reaction monitoring transitions. Detection was achieved by monitoring transitions of 728.0 > 287.1 for Cya-3-*O*-xylrut in ESI+ mode. The analytical conditions were as follows: the ion source was operated at a temperature of 250 °C. The nebulizing gas and nebulizer pressure was 380 Pa. The ion spray voltage was 4500 V. The entrance and focusing potentials were set at 10 and 375 V. The declusterings potential was 80. Analyst software (version 1.4) [29] installed on a Dell computer was used for data acquisition and processing.

#### 3.2.7. Antioxidant Activities Test Methods

##### Folin–Ciocalteu Assay

The total phenolic content of anthocyanins was estimated by a colorimetric assay based on a published procedure [30], with slight modifications. Sample (1 mL) was pipetted into a tube; 1 mL of 50% Folin–Ciocalteu’s reagent and 1 mL of 10% sodium carbonate solution were added. The contents were vortexed for 30 s and allowed to stand at room temperature for 2 h. Absorbance measurements were recorded at 765 nm, using catechins to construct the curves. The results were reported as mean values expressed as mg of catechins equivalents per gram of sample.

##### Ferric Reducing Antioxidant Power (FRAP)

The reducing antioxidant capacity was determined using a modification of the FRAP assay [31]. The FRAP reagent was prepared from 300 mM, pH 3.6, acetate buffer, 20 mM ferric chloride and 10 mM 2,4,6-tripyridyl-*S*-triazine made up in 40 mM hydrochloric acid. All three solutions were mixed together in the ratio of 25:2.5:2.5 (*v*/*v*/*v*). The FRAP assay was performed using reagents preheated to 38 °C. Prior to analysis, the initial absorbance of 3 mL of the reagents, and a 3 mL acetate buffer used as blank, were measured at 593 nm. The samples (100 μL) were transferred into the test tubes containing the reagent. The mixtures were shaken thoroughly and examined after 90 min using the spectrophotometer. The absorbance values at 593 nm were recorded. A higher absorbance indicated a higher ferric reducing power, and the tests were carried out in triplicate. The reducing antioxidant power of the sample was expressed as Trolox equivalent, which was the ratio between the slope of the sample’s regression line and that of Trolox.

##### Free Radical Scavenging Activity (ABTS)

The method of Wojdylo *et al*. [32] was used with slight modification. 2,2′-Azinobis-(3-ethylbenzthiazoline-6-sulphonate) (ABTS) diammonium salt (7 mM) and potassium persulfate (2.45 mM) were mixed and kept in the dark at room temperature for 18 h before use. For this study, the ABTS radical solution was diluted with methanol to an absorbance of 0.7 at 734 nm. The sample solution (0.05 mL) was added to 1 mL of the blue-green ABTS radical solution. The mixture was shaken vigorously and allowed to reach a steady state at room temperature in a dark for 1 min. The decrease in absorbance was measured at 734 nm, and the control consisted of 0.05 mL of Trolox-methanol solution and 1 mL of ABTS radical solution. The tests were carried out in triplicate and the free radical scavenging activity of the sample was expressed as Trolox equivalent.

#### 3.2.8. Statistical Analysis

Statistical analysis of the data was performed by analysis of variance (ANOVA), and the significance of the difference between means was determined by Duncan’s multiple range test (*p* < 0.05) using SAS (Version 8.1, 2000; SAS Inst., Cary, NC, USA). Values are expressed as “mean ± standard deviation”.

#### 3.2.9. Calculation Methods

##### Total Monomeric Anthocyanins (TMA)

Total anthocyanins content was measured using the pH differential method [14], which became a standard AOAC analysis method in 2005, with minor modifications using a UV-2550 UV-Vis spectrophotometer (Shimadzu, Japan). The crude anthocyanins extracts were dissolved in potassium chloride buffer (KCl, 0.025 M, pH 1.0) and sodium acetate (CH=COONa•3H_2_O, 0.4 M, pH 4.5) with a pre-determined dilution factor. The absorbance of the measured samples was read at 510 nm and 700 nm against a blank cell containing deionized water. Results were expressed as mg/mL of equivalent cyanidin-3-glucoside. The absorbance (A) of the diluted sample was then calculated as follows:

(1) A = (A_510nm_ − A_700nm_) pH 1.0 − (A_510nm_ − A_700nm_) pH 4.5

The monomeric anthocyanins pigment concentration in the original sample was calculated according to the following formula:

(2) Anthocyanins content (mg/mL) = A × MW × DF × 1000/(ε × 1)

where the molecular weight of cyanidin-3-glucoside (MW = 449.2), the dilution factor or dilution multiple (DF = 5) and a molar extinction coefficient of cyanidin-3-glucoside of 29,600 (ε = 29,600) [33,34] were used.

##### Adsorption and Desorption Evaluation

The adsorption was evaluated using the following equation:

(3) *Q_e_* = (*C*_0_ − *C_e_*) × *V_i_* × (1 − *M*) × *W*

(4) *D_e_* (%) = *Q_e_*/(*C*_0_ × *V_i_*) × 100%

where *Q_e_* was the adsorption capacity at adsorption equilibrium (µg/g anhydrous resin); *C*_0_ and *C_e_* were the initial and equilibrium concentrations of anthocyanins, respectively (µg/mL); *V_i_* was the volume of the initial sample solution (mL); *M* was moisture content; and *W* was the mass of resin (g); *D_e_* was the adsorption ratio (%).

Desorption was evaluated using the following equations:

(5) *Q_d_* = *C_d_* × *V_d_* (1 − *M*) × *W*

(6) *D_d_* (%) = *Q_d_*/*Q_e_* × 100% = *C_d_* × *V_d_* × (*C*_0_ − *C_e_*) × *V_i_* × 100%

where *Q_d_* was the desorption capacity after adsorption equilibrium (µg/g anhydrous resin); *C_d_* was the concentration of anthocyanins in the desorption solution (µg/mL); *V_d_* was the volume of the desorption solution (mL); *D_d_* was the desorption ratio (%); and *C*_0_, *C_e_*, *V_i_* and *M* were as described above.

##### Adsorption Isotherms

The Langmuir adsorption model can be expressed by the following equation:

(7) *Q_e_* = *Q_m_* × *C_e_*/(*K* + *C_e_*)

where *Q_e_* (µg/g) was the concentration of solute per mass of adsorbent (solid phase), also known as the adsorptive capacity; *C_e_* (µg/mL) was the concentration of solute in solution (liquid phase) at equilibrium; K was the Langmuir constant; and *Q_m_* was an empirical constant.

The Freundlich equation can be expressed as follows:

(8) *Q_e_* = *KC_e_*^1/*n*^

Alternatively, in the form:

(9) ln *Q_e_* = ln *K* + *1/n ×* ln *C_e_*

where *K* was the Freundlich constant, which was an indicator of adsorption capacity; and 1/*n* was an empirical constant related to the magnitude of the adsorption driving force.

##### Purity Evaluation

The purity was evaluated using the following equation:

(10) *P* = *M_A_*/*M_T_* × 100%

where *P* was the purity of anthocyanins (%); *M_A_* was the mass of anthocyanins in the dry products (mg); and *M_T_* was the total mass of dry products (mg).

## 4. Conclusions

In present study, the parameters of a *S. chinensis* anthocyanins purification process by macroporous resin and gelfiltration chromatography were evaluated. The purity of anthocyanins after purification by HPD-300 increased six-fold from 5.08% to 30.43%, and the anthocyanins recovery was 96.5%. The gel-filtration resin used was Bio-Gel P2, and the recycle yield of anthocyanins was 89.24%. The results reflected the advantages of the adsorption–desorption method, which are lower cost, high efficiency, procedural simplicity, and environmental friendliness. The combined method of macroporous resin plus gel filtration chromatography is valuable and practical strategy for separating the high-purity anthocyanins from other herbal plants. The major constituent of *S. chinensis* anthocyanins was cyanidin-3-*O*-xylosylrutinoside as determined by LC-MS. Moreover, the antioxidant activities of *S. chinensis* anthocyanins were investigated. After purification by HPD-300 resin, the total phenolic content of anthocyanins was increased 4.5-fold, while the antioxidant activities of anthocyanins were also increased 1.2-fold (FRAP) and 1.7-fold (ABTS). The results of antioxidant activities study show that *S. chinensis* anthocyanins with notable antioxidant properties for potential use in nutraceutical or functional food formulations as preservatives and colorants.
